# Translational approaches in microbiome research: from functional mechanisms to next-generation therapeutics

**DOI:** 10.3389/fmicb.2026.1943873

**Published:** 2026-09-23

**Authors:** Manoj Kumar, Parul Singh, Noora Almohannadi, Souhaila Al Khodor

**Affiliations:** Microbiome and Biomarkers Discovery Lab, Research Branch, Sidra Medicine, Doha, Qatar

**Keywords:** antimicrobial stewardship, engineered microbial therapies, FMT, gut microbiome, host–microbiome interactions, microbiome-based therapeutics, microbiota modulation, translational microbiome research

## Abstract

Microbiome research has made substantial progress toward clinical translation over the past 2 decades, moving from associative observations toward mechanistic investigation, validation, and therapeutic intervention. This narrative review examines recent advances in the clinical application of microbiome research over the past 5 years, focusing on non-invasive microbiome-derived biomarkers and therapeutic strategies, including personalized probiotics and prebiotics, fecal microbiota transplantation (FMT), and engineered microbial therapeutics, while evaluating the strength of the supporting evidence. Microbiota-based treatments for recurrent Clostridium difficile infection are currently at the forefront of clinical translation, whereas applications in inflammatory, metabolic, neurological, and oncological disorders remain largely investigational. We further examine these translational barriers while highlighting opportunities to integrate microbiome data with systems biology and advanced analytics to improve clinical outcomes and advance precision medicine. By linking mechanistic discoveries with their current level of clinical development, this narrative review critically evaluates the available translational microbiome evidence, identifies key bottlenecks and unresolved knowledge gaps, and outlines priorities for the responsible integration of microbiome-based diagnostics and therapeutics into clinical practice.

## Introduction

The human microbiome comprises diverse communities of bacteria, fungi, archaea, viruses, and their associated genomes, which inhabit and interact with the human body in commensalistic, mutualistic, or pathogenic ways (Grice and Segre, 2011). Under the hologenome framework, the host genome and the collective genomes of resident microbiota are viewed as an integrated system shaping host biology (Rosenberg and Zilber-Rosenberg, 2018). Microbial communities colonize multiple anatomical sites such as the skin, the mammary gland, the mucosa, gastrointestinal, respiratory and urogenital tracts, each adapted to distinct environmental conditions of each niche (Reynoso-García et al., 2022). The gut microbiota comprises a dynamic community of microorganisms that are shaped by the unique physicochemical and nutritional conditions of the intestinal environment (Valencia et al., 2025). The gut microbiome is a major target of microbiome research and therapeutic development because these microbes contribute to vital metabolic, immunological, and physiological processes through intricate interactions with the host (Valencia et al., 2025). Over the past decade, advances in multi-omics technologies and systems biology have revolutionized our understanding of the microbiome, transforming from a passive community to an active regulator of metabolism, immunity, and neuroendocrine function (Cox et al., 2022; Kumar et al., 2020). The functional microbiome is now recognized as an integral component of human physiology, modulating nutrient absorption, energy metabolism, xenobiotic detoxification, and immune maturation (Cox et al., 2022). Conversely, dysbiosis, or imbalance in microbial composition and function, has been linked to a wide range of diseases and pathological conditions including metabolic disorders, inflammatory bowel disease, cancer, and neuropsychiatric conditions (Akobeng et al., 2020; Augustine et al., 2023; Hou et al., 2022; Kumar et al., 2019, 2021b; Saadaoui et al., 2024) among others.

Moving beyond broad mechanistic associations, modern clinical focus has pivoted sharply toward leveraging the microbiome for actionable non-invasive diagnostics and targeted patient risk stratification (Chopra et al., 2024; Kumar et al., 2024; Wu et al., 2024). Metagenomic and metabolomic analyses have revealed disease-specific microbial signatures and metabolite profiles, offering new avenues for non-invasive diagnostics and precision medicine (Philip et al., 2025). For instance, distinct microbial alterations have been linked with esophageal (Lopetuso et al., 2020), gastric (Wang et al., 2020), and oral cancers (Minarovits, 2021; York, 2020), chronic obstructive pulmonary disease (COPD) (Wang et al., 2017) and lung cancer (Ramírez-Labrada et al., 2020) and inflammatory bowel disease (IBD; Alam et al., 2020; Elawad et al., 2022; Galipeau et al., 2021). In some cases, these microbial patterns are not only linked to the presence of disease but also serve as potential indicators of disease onset, remission, progression, or therapeutic response. For example, alterations in the lung and sputum microbiota have been observed during COPD exacerbations (Wang et al., 2016) while shifts in intestinal and stool microbial communities may precede IBD relapse (Elhag et al., 2022; Kumar et al., 2024; Lloyd-Price et al., 2019).

Building on these discoveries, the field of microbial therapeutics is undergoing an extraordinary evolution, transitioning from empirical, whole-ecosystem resets toward precise, rationally designed molecular interventions (Yadav and Chauhan, 2022). Strategies such as microbiota modulation, probiotic and prebiotic supplementation, and fecal microbiota transplantation (FMT) are being explored for their clinical potential (Angoorani et al., 2021; Araujo et al., 2022). More advanced approaches, including the use of defined microbial consortia, genetically engineered gut bacteria, and optimized use of antibiotics, are paving the way toward next-generation microbiome-based therapies (Duncker et al., 2021; Mutalub et al., 2022; Pitashny et al., 2025). Together, these approaches represent a paradigm shift in medicine from treating disease symptoms to modulating the host–microbe ecosystem to restore physiological harmony. By bridging fundamental discoveries with clinical practice, translational microbiome research has the potential to transform approaches to human health and disease management. While several recent reviews have addressed multi-omics, precision microbiome medicine, live biotherapeutic products, and microbiome therapies, few have explicitly looked at how these methods might be incorporated throughout the discovery-to-clinic continuum. In order to bring microbiome-based therapeutics into clinical implementation, this review focuses on the major translational obstacles and potential for combining microbiome research, multi-omics, and precision techniques. We also highlight recent advances in translational microbiome research, focusing on therapeutic strategies, mechanistic insights, and clinical applications. Animal models and *in vitro* systems that offer significant mechanistic or translational insights have been incorporated into our synthesis of information from human research, clinical trials, and clinical-trial registrations. We specifically address three important questions: (1) What degree of evidence supports the use of gut microbiome-derived therapies that have shown clinical efficacy? (2) Which human studies have confirmed the microbiome-host pathways found in preclinical research? and (3) What methodological, clinical, manufacturing, and regulatory obstacles still hinder the translation? By answering these queries, we hope to pinpoint important gaps in the existing body of knowledge and draw attention to new approaches that could help create microbiota-based treatments that are safe, efficient, and clinically scalable.

## Methodology

This narrative review evaluated the clinical translation of microbiome-based diagnostics and therapeutics. PubMed/MEDLINE, Embase, JSTOR and ClinicalTrials.gov were searched from database inception through 15 May 2026 using combinations of microbiome- and therapy-related terms, including microbiome, microbiota, probiotics, prebiotics, FMT, microbial consortia, engineered microorganisms, and bacteriophages. English-language preclinical and clinical studies, observational studies, trials, systematic reviews, and meta-analyses providing relevant mechanistic or translational evidence were considered. Given the substantial heterogeneity of interventions, clinical indications, and study designs, no quantitative meta-analysis was performed. The evidence was evaluated narratively, with particular attention to clinical relevance, evidence attribution, translational limitations, and balanced interpretation of the findings.

## From taxonomy to function: microbial metabolites in host health and disease

A defining paradigm shift in contemporary microbiome science is the combination of the taxonomic, strain-level, functional, transcriptional, and metabolomic data to comprehend the interactions between microbial communities and their hosts. While functional and molecular techniques offer complimentary information on the genes, pathways, activities, and metabolites associated with these communities, taxonomic profiling offers crucial information on community makeup and organismal identity (Hou et al., 2022; Kumar et al., 2026). Microbial communities mediate a wide range of physiological functions, including immune system development and nutrient biosynthesis (Clemente et al., 2012; Hsu and Schnabl, 2023; Kumar et al., 2020, 2021a). These effects can be mediated through microbial metabolites and enzymes that produce, modify, or modulate bioactive molecules and subsequently influence host receptors and signaling pathways (Kim et al., 2024). Microbiome-associated metabolites can also be broadly distinguished according to their origin and mode of generation. Some compounds, such as short-chain fatty acids (SCFAs) such as butyrate and propionate are produced primarily through the microbial fermentation of dietary fibers (Tan et al., 2014; Xiong et al., 2022) are essential in modulating immune responses, preserving epithelial barrier integrity, and regulating host energy metabolism. Others are host-derived molecules that are subsequently modified by microbial enzymes such as vitamins B12 (Piwowarek et al., 2018) and K (Ellis et al., 2021), neurotransmitters like gamma-aminobutyric acid (GABA), and key serotonin precursors, as well microbial enzymes that actively biotransform host-derived primary bile acids into distinct secondary bile acid derivatives, which regulate systemic lipid and glucose metabolism (Ashique et al., 2024; Elhag et al., 2021; Kim et al., 2024). However, microbial transformation within the intestine does not necessarily indicate systemic exposure or a clinically meaningful biological effect, and the magnitude and relevance of these effects depend on absorption, circulation, tissue distribution, and host metabolism (Jyoti Dey, 2025; Li et al., 2026). Conversely, microbial dysbiosis can result in altered taxonomic composition, changes in metabolic output, and alterations in secreted vesicles. These imbalances have been strongly linked to a wide spectrum of diseases, including IBD, obesity, type 2 diabetes, cancer, and neurological disorders (Chopra et al., 2022; Iliev et al., 2025; Kumar et al., 2019; Loh et al., 2024; Nobels et al., 2025; Takahashi et al., 2016; Zhang et al., 2024), thereby underscoring the microbiome's critical functional relevance and its emerging role as a target for diagnostics and therapeutics. Associations between microbiome-derived metabolic features and disease do not by themselves establish causality, understanding microbiome function requires integration across taxonomic identity, strain-level genomic capacity, gene expression, enzymatic activity, metabolite production or transformation, downstream signaling, and physiological or clinical outcomes. The experimental context should be taken into consideration when interpreting mechanistic evidence, with results from cell culture and *ex vivo* systems offering mechanistic insight, animal models allowing evaluation of host-level effects, human observational studies identifying correlations, and controlled intervention studies offering stronger evidence for causal and translational relevance.

## Reshaping the gut microbiota: the synergistic effects of nutrition and physical activity

Because the functional output of the microbiota is closely linked to dietary substrates and host lifestyle, nutrition and physical activity represent accessible, non-pharmacological strategies for modulating microbial composition and function, providing a potential bridge between lifestyle modification and microbiome-directed precision medicine (Singh et al., 2019; Zeng et al., 2025). High-fiber, plant-rich diets have been associated with enrichment of fiber-degrading bacteria such as *Prevotella* and *Xylanibacter*, supporting SCFA production (De Filippo et al., 2010; Jamieson et al., 2023; Medawar et al., 2019), while Mediterranean dietary patterns have been associated with increased *Bifidobacterium* and reduced inflammatory markers like C-reactive protein and IL-6 (Haskey et al., 2023; Perrone and D'Angelo, 2025). However, these microbial responses are highly dependent on host characteristics and environmental context and may represent short-term changes rather than durable ecological restructuring.

Physical activity has similarly been associated with functionally beneficial microbial taxa, including the mucin-degrading *Akkermansia muciniphila* and butyrate-producing *Roseburia hominis* (Hughes, 2020; Mohr et al., 2020), while athlete studies have reported differences in microbial diversity and metabolic capacity compared with sedentary individuals (Barton et al., 2018; Clarke et al., 2014). These associations may also be influenced by diet, body composition, medication use, and training intensity. Thus, nutrition and exercise are best considered complementary strategies for modulating the microbiome, without assuming an inherently synergistic effect. Controlled longitudinal studies with standardized dietary and exercise protocols are needed to establish causality, durability, and clinical benefit, before these strategies can be reliably incorporated intopersonalized microbiome-based interventions.

## Microbial biomarkers and metabolomics: advancing non-invasive diagnostics through multi-omics integration

Advances in functional microbiome research are enabling the translation of microbial signatures and metabolites into non-invasive diagnostic, prognostic, predictive, and treatment-response biomarkers. Microbial metabolites, including SCFAs, BCAAs, bile acids, TMAO, tryptophan derivatives, imidazole propionate, as well as microbial components such asmicrobial endotoxins (LPS/LOS), provide functional links between microbial activity and host physiology and have been associated with metabolic, inflammatory, cardiovascular, neurological, and gastrointestinal disorders (Agus et al., 2021; Arya et al., 2025; Batch et al., 2014; Hajjo et al., 2022; Neves et al., 2015; Perl et al., 2025; Sivaprakasam et al., 2016; Zeng et al., 2025). However, as highlighted above, these biomarkers are often context-dependent and influenced by diet, medications, geography, host characteristics, sampling, and analytical procedures.

Integration of metagenomics, metatranscriptomics, proteomics, and metabolomics provides an opportunity to connect microbial composition with functional outputs and identify clinically relevant signatures (Chetty and Blekhman, 2024; Kumar et al., 2021c). Machine learning can further support biomarker discovery and patient stratification, although robust external validation is required to minimize overfitting, batch effects, and population-specific bias. Importantly, multi-omics models should demonstrate added clinical value over established biomarkers while considering cost and analytical complexity. Thus, translating microbial associations into clinically actionable biomarkers requires independent and prospective validation, standardized analytical workflows, and assessment of diagnostic or predictive performance, these steps are essential for bridging functional microbiome research with precision diagnostics and guiding the development of next-generation microbial therapeutics.

## The emerging landscape of microbiome-based therapeutics

Advances in mechanistic microbiome research have supported the development of therapeutic strategies aimed at modifying microbial communities or their functions. Preclinical and clinical studies suggest that microbiota modulation can influence colonization resistance, host immunity, and therapeutic responses; however, the strength of evidence varies substantially across diseases and interventions (Gilbert et al., 2025). Rather than assuming a universal “healthy microbiome,” therapeutic approaches increasingly aim to restore or modify specific microbial functions and disease-associated ecological features.

The clinical maturity of these approaches also differs considerably. Microbiota-based therapies for recurrent *Clostridioides difficile* infection currently have the strongest clinical and regulatory evidence (Berry and Khanna, 2023), whereas conventional dietary interventions and probiotics remain at varying stages of clinical investigation (Aslam et al., 2026). Similarly, associations between the gut microbiome and treatment responses, including immune checkpoint inhibitor therapy, have generated important therapeutic hypotheses, but broader clinical implementation requires further prospective validation (Zhao et al., 2023). As illustrated in Figure 1, these emerging clinical interventions span a sophisticated technological continuum. They range from simple, targeted dietary and lifestyle restructurings to the empirical administration of standard commercial probiotics, the manufacturing of synthetically designed microbial consortia, and advanced synthetic biology approaches utilizing genetically engineered living medicines and specialized bacteriophage platforms. Although these therapeutic modalities should therefore be viewed as distinct strategies with different mechanisms, levels of evidence, manufacturing requirements, and regulatory pathways. This therapeutic continuum is rapidly evolving, bringing with it unique mechanistic advantages, distinct operational hurdles, and unprecedented opportunities for precision, ecosystem-preserving interventions across human healthcare.

**Figure 1:**
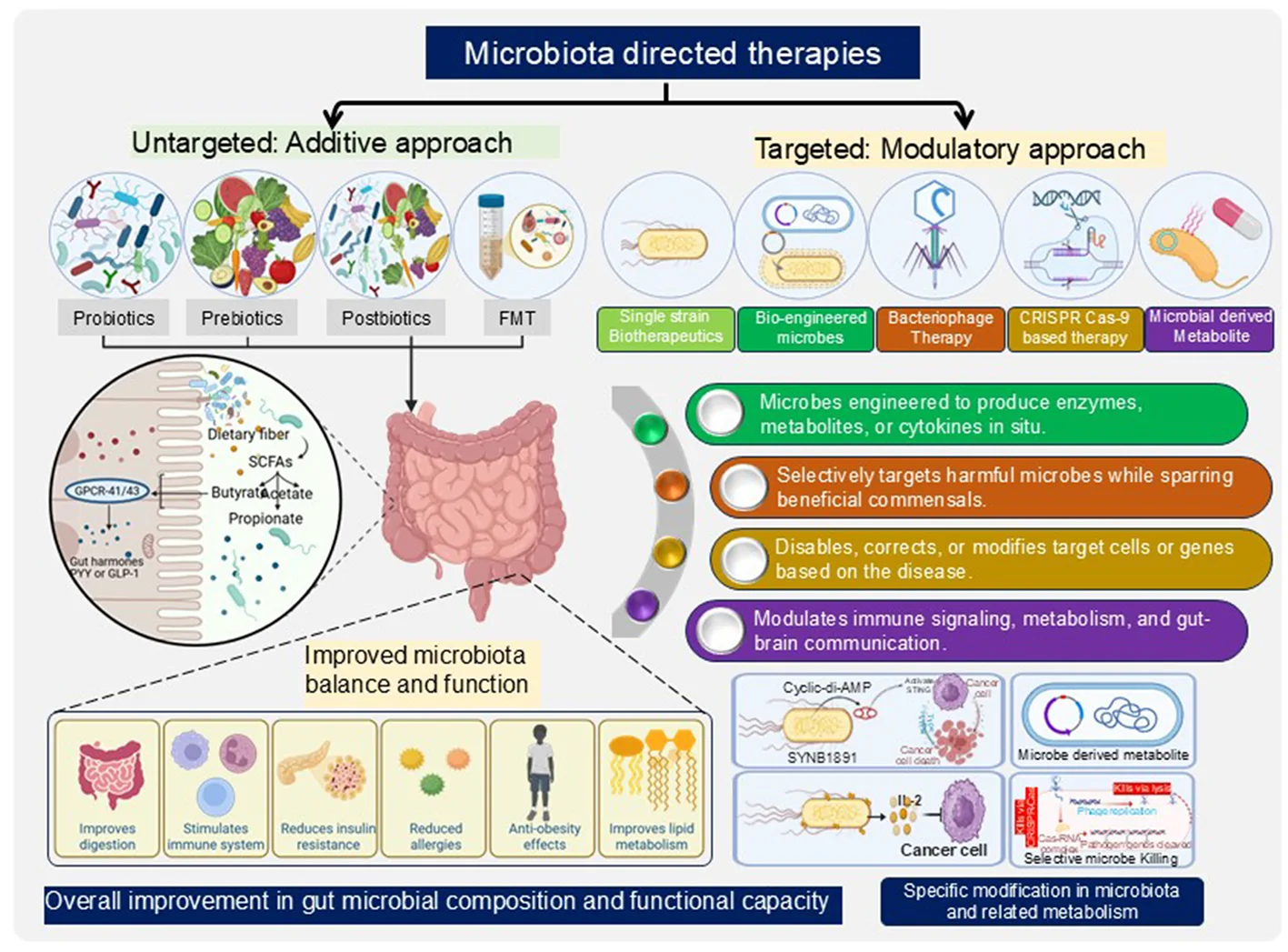
*Microbiota-directed therapeutic strategies in human disease*. Accumulating evidence from epidemiological and experimental studies highlights the gut microbiome as a key regulator of metabolic health, driving interest in both untargeted and targeted microbiota-directed therapies for various diseases. *Untargeted approaches* include probiotics, prebiotics, postbiotics, and fecal microbiota transplantation, which broadly modulate microbial community composition and function. Established probiotics such as Bifidobacterium, Lactobacillus, and Saccharomyces spp. have long records of safety, while next-generation candidates include *E. coli* Nissle 1917, *Akkermansia muciniphila*, and selected Clostridia spp. Fermentation of dietary fiber and nutrients generates short-chain fatty acids (acetate, propionate, and butyrate), which signal *via* GPCR41/43 and stimulate PYY and GLP-1, improving digestion, immune tone, insulin sensitivity, lipid metabolism, allergy control, and intestinal barrier integrity. *Targeted approaches* aim to modulate specific microbial or host pathways using precision strategies, including single-strain live biotherapeutics, bioengineered microbes, bacteriophage therapy, CRISPR–Cas-based interventions, and microbially derived metabolites. These modalities achieve therapeutic effects through *in situ* delivery of bioactive factors, selective pathogen depletion, gene or cellular editing, and modulation of immune and metabolic pathways, enabling precise and programmable microbiota and metabolic remodeling.

## The clinical landscape of probiotic and prebiotic therapies

Probiotics and prebiotics represent accessible and widely studied strategies for modulating the gut microbiota and host functions (Huang et al., 2025). Probiotics are live microorganisms that may confer health benefits through strain-specific mechanisms, including modulation of immune responses, enhancement of epithelial barrier function, competitive inhibition of pathogens, and regulation of microbial metabolites (Suez et al., 2019). Similarly, prebiotics such as inulin, fructooligosaccharides (FOS), and galactooligosaccharides (GOS) selectively fuel host microbes to produce functional metabolites like SCFA (Ansari et al., 2023), indirectly enhance host immune defenses and suppress pathogens to support overall intestinal homeostasis (Maier et al., 2017; Shoaf et al., 2006). However, clinical responses to both interventions are highly dependent on the strain, dose, formulation, indication, and host characteristics, contributing to substantial variability across studies (Zandifar et al., 2025). This context dependency is also evident at the metabolite level; for example, while butyrate has demonstrated tumor-suppressive effects in some settings (Sun et al., 2024), it may promote tumorigenesis under specific genetic conditions (Belcheva et al., 2014).

Despite this clinical variability, in clinical settings, probiotics and prebiotics have been evaluated across gastrointestinal and metabolic disorders (Table 1), including IBD (Vallejos et al., 2025), IBS (Lopes et al., 2024), and CRC (Gan et al., 2025), although their clinical utility depends strongly on the specific intervention and intended endpoint. Among gastrointestinal indications, selected high-potency multi-strain formulations, historically studied as VSL#3, have shown benefits in preventing pouchitis and maintaining remission in ulcerative colitis (Gionchetti et al., 2003), though these benefits do not replicate in Crohn's disease (Limketkai et al., 2020). For symptom management, specific synbiotics containing *Lactobacillus* and *Bifidobacterium* strains with short-chain FOS (NCT04206410) have shown modest improvements in bowel habits and flatulence in diarrhea-predominant IBS (Skrzydło-Radomańska et al., 2020), despite broader meta-analyses in IBS and NSAID-induced enteropathy remaining inconsistent (Moayyedi et al., 2010; Montalto et al., 2010). For reducing treatment-related toxicity, multi-strain combinations (*B. breve, B. infantis, B. longum, L. acidophilus*) have demonstrated efficacy in mitigating chemotherapy-induced diarrhea and mucositis (Hu et al., 2015; Kahouli et al., 2017). In contrast, exploratory trials evaluating *in situ* prebiotic generation *via* transglucosidase for glycemic control in type 2 diabetes (Sasaki et al., 2013) or *Bifidobacterium*- and *L. fermentum*-enriched dairy for cognitive function (Kim et al., 2021; Park et al., 2020); represent early surrogate findings that lack replication in adequately powered phase three studies.

**Table 1:** Emerging microbial-based therapies for human therapeutic use.

Intervention/sponsor	Type	Therapeutic use	Status/phase	References
FMT—Oslo University Hospital	Microbial therapy	FMT for Stage IV Melanoma cancer patients not responding to ICI therapy	Phase 2	NCT05286294
FMT—The Netherlands Cancer Institute	Microbial therapy	FMT for Stage III and IV Melanoma cancer patients not responding to ICI therapy	Phase 1/2	NCT05251389
FMT—Asan Medical Center	Microbial therapy	FMT with nivolumab for Solid Carcinoma	Phase 1	NCT05533983
FMT—University of Pittsburgh	Microbial therapy	hdFMT for lung cancer patients with relapsed/refractory PD-L1	Phase 2	NCT05669846
FMT—Canadian Cancer Trials Group	Microbial therapy	LND101 for immune checkpoint blockade in advanced melanoma (Canbiome2)	Phase 2	NCT06623461
FMT—Assistance Publique—Hôpitaux de Paris	Microbial therapy	FMT for melanoma patients treated with CTLA-4 and PD-1 Inhibitors	Phase 2	NCT04988841
FMT—Gateway Foundation for Cancer Research	Microbial therapy	hdFMT for PD-1 refractory advanced melanoma	Phase 2	NCT06030037
FMT—Fujian Provincial Hospital	Microbial therapy	FMT for hepatocellular carcinoma non-resectable	Phase 1	NCT06643533
FMT—Michael Dill	Microbial therapy	FMT for hepatocellular carcinoma and Immunotherapy	Phase 2	NCT05690048
FMT—Medical University of Vienna	Microbial therapy	FMT for hepatocellular carcinoma	Phase 2	NCT05750030
FMT—John Lenehan	Microbial therapy	FMT for Metastatic Advanced Pancreatic Ductal Adenocarcinoma	Phase 1	NCT06393400
FMT—M.D. Anderson Cancer Center	Microbial therapy	FMT for Pancreatic Ductal Adenocarcinoma	Phase 1	NCT04975217
FMT—Changhai Hospital	Microbial therapy	Synergy with advanced gastric cancer treatment	Phase 1	NCT06346093
FMT—Shenzhen Hospital of Southern Medical University	Microbial therapy	FMT for colorectal adenomas	Phase 2	NCT06205862
FMT—Genton Graf Laurence	Microbial therapy	FMT for pancreatic cancer	Observational	NCT05606523
FMT—M.D. Anderson Cancer Center	Microbial therapy	FMT for immune-related diarrhea/colitis.	Phase 1	NCT04038619
FMT—University of Aarhus	Microbial therapy	FMT for immune checkpoint inhibitor-mediated diarrhea/colitis	NA	NCT06206707
FMT—M.D. Anderson Cancer Center	Microbial therapy	FMT for melanoma or genitourinary cancer	Phase 1	NCT03819296
Prebiotics—M.D. Anderson Cancer Center	Dietary intervention	Dietary supplement for resectable melanoma initiating neoadjuvant and ICB therapy	Phase 2	NCT06548789
Pre/Pro/Postbiotics—M.D. Anderson Cancer Center	Dietary intervention	Prebiotic for gut microbiome in melanoma patients starting ICB therapy	NA	NCT06466434
Prebiotics—Center hospitalier de l'Université de Montréal	Dietary intervention	Prebiotic for gut microbiome in metastatic melanoma patients starting ICI therapy	Phase 1	NCT05303493
Prebiotics—M.D. Anderson Cancer Center	Dietary intervention	Prebiotic for gut microbiome in patients with ICI-refractory melanoma	Phase 2	NCT06250335
Prebiotics—Fondazione del Piemonte per l'Oncologia	Dietary intervention	Prebiotic Inulin on Patients with head and neck squamous cell carcinoma treated with ICI	NA	NCT05821751
Probiotics—Necmettin Erbakan University	Microbial intervention	*Bifidobacterium animalis lactis* BL-04 on the effect-tiveness of immunotherapy in patients with metastatic	NA	NCT06428422
Probiotics—Rise Therapeutics LLC	Microbial intervention	R-5780 in patients with Solid Tumors treated with PD-1	Phase 1	NCT06398418
Pre/Probiotics—AC Camargo Cancer Center	Dietary/microbial intervention	Pre-and probiotics with Ch-RT treatment for patients with anal canal squamous cell carcinoma	Phase 2	NCT03870607
Probiotics—Zhejiang University	Microbial intervention	Probiotics with targeted therapy in patients with metastatic colorectal cancer	NA	NCT04131803
Probiotics—Qingdao University	Microbial intervention	Probiotics with chemotherapy in gastric cancer	NA	NCT05901779
Probiotics—National University Hospital, Singapore	Microbial intervention	Perioperative probiotics in colorectal cancer surgery	NA	NCT06456229
Probiotics—The Netherlands Cancer Institute	Microbial intervention	SMT04 for advanced adenoma recurrence	NA	NCT05592886
Probiotics—Mayo Clinic	Microbial intervention	VSL#3^®^ 450B in patients receiving immunotherapy	NA	NCT06508034
Postbiotics—Federal University of Minas Gerais	Dietary/microbial intervention	Synbiotic for Patients Undergoing Chemotherapy in Cancer	NA	NCT06576986
Probiotics—University Hospital, Ghent	Microbial intervention	Prevent endocrine therapy-related vulvo-vaginal atrophy	Phase 4	NCT05562518

FMT, fecal microbiota transplantation; hdFMT, high-dose fecal microbiota transplantation; ICI, immune checkpoint inhibitor; PD-1: programmed death-1; PD-L1: programmed death-ligand 1; CTLA-4: cytotoxic T-lymphocyte–associated protein 4; ICB, immune checkpoint blockade; NA, not applicable.

Beyond these clinical applications, experimental studies have broadened the potential biological scope of probiotic interventions, although their direct translational relevance remains uncertain. In rodent models, *Lactobacillus plantarum* 299v and *Lactobacillus rhamnosus* GR-1 showed cardioprotective effects following experimental ischemic injury (Gan et al., 2014), while *Lactobacillus acidophilus* TW01 attenuated air-pollution-induced lung injury (Luo and Chen, 2025). These findings provide preclinical mechanistic evidence supporting the investigation of additional therapeutic applications; however, validation in well-designed human studies is required before conclusions regarding clinical efficacy can be drawn.

Probiotics and prebiotics are generally well tolerated; however, rigorous safety oversight remains essential because adverse events are frequently underreported in clinical trials, and rare but serious complications, including bacteremia, *Saccharomyces boulardii* fungemia, and endocarditis, have been reported, particularly in immunocompromised or intestinal barrier-compromised patients (Bafeta et al., 2018). Moving forward, clinical integration will require strain-specific safety and efficacy validation, standardized adverse-event reporting, and stringent manufacturing and quality-control measures to minimize microbial misidentification, contamination, and the potential transmission of antimicrobial-resistance genes.

In rodent models, *Lactobacillus plantarum* 299v significantly reduced infarct size and improved cardiac function following ischemia-reperfusion injury, while *Lactobacillus rhamnosus* GR-1 produced similar effects in myocardial ischemia (Gan et al., 2014). Additionally, Probiotic modulation of the gut microbiota has also been investigated in pulmonary disease models; for example *Lactobacillus acidophilus* TW01 can attenuate air pollutant-induced lung injury and enhance respiratory defenses (Luo and Chen, 2025). However, despite these therapeutic prospects, safety concerns remain. A comprehensive systematic review encompassing 384 randomized controlled trials revealed that adverse event reporting in probiotic literature is profoundly inadequate, especially in immunocompromised or gut-barrier-impaired patients. Case reports have linked probiotic use to serious infections, including bacteremia, fungemia, and endocarditis (Bafeta et al., 2018), highlighting the need for rigorously designed clinical trials with comprehensive safety evaluations before broader clinical adoption. Positioned at the intersection of nutrition, microbiology, and medicine, prebiotic and probiotic-based therapies hold undeniable promise for both disease prevention and targeted therapeutic intervention, provided their clinical deployment is guided by precision and stringent safety validation.

## Fecal microbiota transplantation and next-generation microbial consortia

When conventional microbiome-directed approaches such as probiotic and prebiotic supplements, are insufficient to restore a severely disrupted intestinal ecosystem, FMT provides a more extensive microbiota-restoration strategy. FMT involves the transfer of processed fecal microbiota from carefully screened healthy donors into the gastrointestinal tract of recipients, has emerged as an effective microbiota-based highly disruptive therapeutic strategy approach (Borody et al., 2013). Although its precise molecular and cellular mechanisms remain incompletely understood, clinical studies have reported resolution rates of approximately 85–90% following repeated FMT for rCDI, supporting its established clinical role in appropriately selected patients (Fischer et al., 2017; Tariq et al., 2023; van der Sligte et al., 2026). The clinical success of microbiota restoration in rCDI has also supported the development of manufactured microbiota-based products. REBYOTA (Lee et al., 2023) (Ferring Pharmaceuticals) and VOWST (Feuerstadt et al., 2022; Seres Therapeutics) are FDA-approved specifically for the prevention of rCDI (Sims et al., 2023). REBYOTA is a rectally administered fecal microbiota suspension, whereas VOWST is an orally administered preparation of purified fecal microbiota spores. Although these products differ from conventional donor-dependent FMT in their composition, manufacturing, quality control, pharmacological characterization, and regulatory pathways.

Beyond rCDI, FMT is increasingly being investigated for diverse conditions, including inflammatory disorders, metabolic syndrome, oncology, graft-vs.-host disease (GVHD), and neurodevelopmental conditions; however, these applications remain investigational. MaaT013, a standardized pooled-donor microbiota product, is being evaluated for acute corticosteroid-refractory GVHD in patients non-responsive to first-line immunotherapy (Malard et al., 2023). The open-label Phase 3 trial reported encouraging interim results in January 2025, with a 62% response rate (NCT05017688, NCT04769895). Similarly, SER-155, a defined investigational microbial consortium for patients undergoing allogeneic hematopoietic stem-cell transplantation, has shown preliminary evidence of reduced bloodstream infections (10.0%) compared to the placebo cohort (42.9%), underscoring the therapeutic potential of microbiome modulation beyond gastrointestinal disorders (Ponce et al., 2025).

FMT has also been investigated as an adjunct to immune-checkpoint inhibition in patients with melanoma resistant to anti-PD-1 therapy. Early clinical studies suggest that donor-derived FMT may restore treatment responsiveness in a subset of patients and is associated with changes in gut microbiota and systemic and tumor immune responses (Baruch et al., 2021; Davar et al., 2021). This clear clinical benefit correlated directly with a marked expansion and activation of cytotoxic CD8+ T cells within the tumor microenvironment, coupled with a simultaneous, profound depletion of highly immunosuppressive, IL-8-expressing myeloid cells. These findings are promising but require confirmation in larger controlled trials. Likewise, small open-label studies have explored FMT in children with autism spectrum disorder (ASD), but the absence of appropriate control groups, small sample sizes, subjective outcomes, developmental variability, and potential placebo effects preclude conclusions regarding efficacy; these observations should therefore be considered preliminary (Li et al., 2021).

FMT is generally well tolerated in appropriately screened recipients, but infectious transmission and other adverse events remain important considerations, particularly in immunocompromised individuals (Shogbesan et al., 2018). Additional limitations include donor availability, donor-to-donor variability, screening requirements, variable engraftment, and lack of standardized protocols for preparation, administration, and follow-up (Park and Seo, 2021). Consequently, its use beyond established rCDI indications requires further controlled clinical evaluation.

## The paradigm shift toward defined microbial consortia and live biotherapeutic products

These limitations have encouraged the development of more standardized microbiota-based approaches, including cultured and rationally designed microbial consortia (Gilbert et al., 2016; Louie et al., 2023). These approaches should not be considered a linear progression from FMT, as they differ substantially in composition, biological complexity, manufacturing, quality control, and regulatory requirements. Defined microbial consortia may reduce donor-dependent variability and the risk of pathobiome transmission (Tseng et al., 2025), although challenges related to strain stability, antimicrobial-resistance genes (Honda et al., 2023), manufacturing consistency, engraftment, and long-term safety remain. Consequently, a well-funded, rapidly expanding sector of the global biotechnology and pharmaceutical industry is heavily investing in the rational design of defined consortia, driving a major pharmacological paradigm shift toward scalable, standardized, and mechanistically defined microbiome therapeutics capable of consistent, industrial-scale manufacturing.

Early proof-of-concept studies demonstrated that a simplified cocktail of 10 bacterial species could successfully eradicate *C. difficile* (Tvede and Rask-Madsen, 1989). More recently, VE303, a defined bacterial consortium of eight commensal Clostridia strains, significantly reduced rCDI risk compared to, although validation in a larger phase three cohort remains necessary (Louie et al., 2023). A recent review offers perspectives on ongoing clinical trials of defined microbial consortia and outlines the current clinical development (Gilbert et al., 2025; Table 1). Moving forward, the optimization and rational engineering of these synthetic platforms will increasingly rely on advanced computational biology, predictive mathematical modeling, and high-throughput multi-omics integrations. These digital frameworks will allow researchers to accurately map, simulate, and stabilize the intricate metabolic and ecological networks within therapeutic consortia, ensuring predictable, highly reproducible clinical outcomes.

## Synthetic biology and next-generation engineered programmable probiotics

The past decade has seen a surge of interest in using synthetic biology to genetically modified microbes for targeted disease treatment (Kim et al., 2023). Unlike traditional probiotics or FMT, these engineered microbes are programmed with novel “sense-and-respond” genetic circuits: for example, nonpathogenic strains can be designed to detect disease signals in the gut and produce therapeutic or immune-modulatory molecules *in situ* (Charbonneau et al., 2020). By definition, engineered bacteria are microbes that have been genetically modified to perform a specific diagnostic or therapeutic function (Kim et al., 2023). Under this paradigm, non-pathogenic bacterial chassis are engineered to detect distinct chemical signals of active disease within the complex gut lumen—such as localized inflammatory biomarkers, oxidative stress molecules, or specific pathogen-derived quorum-sensing keys. Upon circuit activation, these living medicines are designed to produce therapeutic molecules locally, potentially increasing exposure at the intended site while limiting systemic effects. This paradigm of programmable living medicines is rapidly advancing toward clinical translation, supported by an evolving regulatory framework. Table 2 summarizes engineered microbial therapeutics across different stages of preclinical and clinical development, including their chassis, therapeutic functions, indications, and developmental status.

**Table 2:** Engineered bacterial therapeutics in preclinical and clinical development.

Engineered bacteria/company	Type	Therapeutic use	Status	References
*Lactobacillus jannaschii* (cyanovirin-N)	Engineered strain	HIV prevention	Preclinical	Grice and Segre, 2011
*Lactococcus lactis* (IL-10)	Engineered strain	CD	Preclinical	Rosenberg and Zilber-Rosenberg, 2018
*Lactococcus lactis* (IL-2)	Engineered strain	Cancer immunotherapy	Preclinical	Reynoso-García et al., 2022
*Lactococcus lactis* AG013 (Trefoil Factor 1)/Oragenics	Engineered strain	Oral mucositis	Phase 2b	Cox et al., 2022; Valencia et al., 2025
*L. lactis* GEN-001 (+ pembrolizumab ± mFOLFOX)/Genome and Company	Single strain	Biliary tract cancer (advanced refractory)	Phase 2	NCT05998447
*L. lactis* GEN-001 (+ avelumab)/Merck and Co., Pfizer, Genome and Company	Single strain	Gastric cancer (PD-L1 positive)	Phase 2	NCT05419362
*L. gasseri* KBL697/KoBioLabs	Engineered strain	Biliary tract cancer	Phase 2	NCT04911751
*L. cremoris* AUP-16/Aurealis pharma	Single strain (GMO)	Diabetic foot ulcer	Phase 2	NCT06111183
*E. coli Nissle* 1917 (IL-10)/Synlogic therapeutics	Engineered strain	IBD	Phase 1	Kumar et al., 2020
E. coli Nissle 1917 (SYNB1934)/Synlogic therapeutics	Engineered strain	Phenylketonuria (PKU)	Phase 3	NCT05925174
E. coli Nissle 1917 (SYNB1891)/Synlogic therapeutics	Engineered strain (STING agonist)	Cancer (solid tumors)	Phase 1	Hou et al., 2022
E. coli Nissle 1917 (SYNB1353)/Synlogic therapeutics	Engineered strain	Homocystinuria (HCU)	Phase 1	NCT05462132
E. coli Nissle 1917 (SYNB8802)/Synlogic therapeutics	Engineered strain	Enteric hyperoxaluria	Phase 1b	NCT04629170
NOV-001/Novome biotechnologies	Engineered strain	Hyperoxaluria	Phase 1	NCT04909723
*Salmonella typhimurium* (TNF-α)	Engineered strain	Cancer	Phase I	Kumar et al., 2019
*Clostridium novyi*-NT/BioMed Valley Discoveries, Inc	Anaerobic spore	Hypoxic tumors	Phase I	NCT01924689
Synbiotic (undisclosed)/Leeuwenhoek laboratories co.	Consortium + prebiotic	Obesity	Phase 2	NCT06578143
*B. animalis subsp. lactis* B94/Lallemand health solutions	Single strain	Infantile colic	Phase 2	NCT06385054
*A. muciniphila* p2261/EverImmune	Single strain	Non-small cell lung or renal cell carcinoma	Phase 2	NCT05865730
BGY-1601-VT (*L. rhamnosus* Lcr35)/Nexbiome therapeutics	Single strain	Bacterial vaginosis (BV), Vulvovaginal Candidiasis	Phase 2	NCT06450990
ILP100-Topical (emilimogene sigulactibac)/Ilya Pharma	Single strain	Diabetic foot ulcer	Phase 2	NCT05608187
ADXS11-001 *L. monocytogenes* (ADXS-HPV)/Advaxis Inc.	Engineered strain immunotherapy (live vaccine)	HPV-associated cancers	Phase 2	NCT02002182
*L. monocytogenes* (ADXS-PA)/Advaxis Inc.	Engineered strain immunotherapy (live vaccine)	Metastatic prostate cancer	Phase 2	Valencia et al., 2025
*B. longum* (APS001F)/Anaeropharma Science	Engineered strain	Solid tumors (cancer)	Phase 1	NCT01562626
*S. epidermidis* (AZT-04)/Azitra Inc	Engineered strain	Cancer therapy-associated rashes	Phase 1	NCT03820076
*S. Typhi* Ty21a (VXM01)/VAXIMM AG	Engineered strain immunotherapy (live vaccine)	Glioblastoma (VEGFR2-targeting)	Phase 2	NCT02718443
*Bacillus velezensis* ADS024/Adiso Therapeutics	Single strain	CDI	Phase 1	NCT04891965
BMC128 (immune checkpoint inhibitors)/Biomica	Defined consortium (4 strains)	Solid tumors (unspecified)	Phase 1	NCT05354102
SER-155/Seres therapeutics	Defined consortium (16 strains)	Diarrhea in People on Immunotherapy	Phase 1	NCT06801067
*Faecalibacterium prausnitzii* and *Desulfovibrio piger*/MetaboGen AB	Defined consortium (2 strains)	Diabetic	Interventional	NCT04938843
*Bacteroides fragilis* (Checkpoint modulator)	Engineered strain	Cancer immunotherapy	Preclinical	

CD, Crohn's disease; GMO, genetically modified organism; IBD, inflammatory bowel disease; PKU, phenylketonuria; HCU, homocystinuria; STING, stimulator of interferon genes (agonist); TNF-α: tumor necrosis factor-alpha; HPV, human papillomavirus; VEGFR2, vascular endothelial growth factor receptor 2; CDI, clostridioides difficile infection; BV, bacterial vaginosis; IL-10, interleukin-10; HIV, human immunodeficiency virus; PD-L1: programmed death-ligand 1.

One of the earliest and most advanced applications of microbial therapeutics has been in correcting inborn errors of metabolism. Engineered *E. coli* Nissle 1917 has been investigated as a chassis for metabolic disorders, including SYNB1618 and its optimized successor SYNB1934 for phenylketonuria (PKU; Charbonneau et al., 2021), SYNB1353 for homocystinuria (Perreault et al., 2024), and SYNB8802 for enteric hyperoxaluria (Lubkowicz et al., 2022). However, these programs have demonstrated variable clinical progress and should not be regarded as uniformly successful. Although SYNB1934 progressed from Phase 2 into a pivotal Phase 3 study, that study was subsequently discontinued after interim analysis indicated that it was unlikely to meet its primary endpoint (NCT05764239). SYNB1891 has also been evaluated as a STING-activating engineered bacterial therapy for cancer immunotherapy (Luke et al., 2023), while SYNB1020 for hyperammonemia (Kurtz et al., 2019), SYNB2081 for gout (Dalbeth et al., 2019) and other candidates further illustrate the broad range of indications explored using engineered bacterial platforms.

Expanding beyond the traditional *E. coli* Nissle chassis, synthetic biologists are exploring alternative gut commensals for therapeutic applications. Engineered *Lactococcus lactis* strains expressing IL-10 or trefoil factors have been evaluated for intestinal inflammation and mucosal repair (de Moreno de Leblanc et al., 2011; Yin et al., 2025), while genetically modified *Listeria monocytogenes* and *Bifidobacterium* strains have been investigated as vectors for cancer-directed therapeutics. For example, ADXS11-001 (ADXS-HPV), an attenuated *L. monocytogenes* strain, was engineered to express a fusion of listeriolysin O with the HPV-16 E7 antigen (Galicia-Carmona et al., 2021), whereas APS001 uses recombinant *Bifidobacterium longum* expressing *E. coli* cytosine deaminase (NCT01562626; Shioya et al., 2021). Engineered microorganisms are also being explored for topical and antimicrobial applications, including engineered *Staphylococcus epidermidis* (Madaan et al., 2024) and CRISPR-based *Lactobacillus plantarum* approaches (Liu et al., 2023). These candidates differ substantially in microbial chassis, therapeutic payload, mechanism, delivery route, indication, and stage of clinical development. Novel oral, intratumoral, and topical delivery strategies are also being investigated for gastrointestinal cancers, glioblastoma, and diabetic foot ulcers (Tables 1, 2). Collectively, these studies demonstrate the broad therapeutic potential of engineered microbial platforms, although their clinical maturity varies considerably and most applications remain investigational.

## Balancing infection control and microbiota preservation: role of antibiotics

The development of microbiome-directed therapies highlights a broader requirement in translational medicine: preserving the host–microbial ecosystem while effectively controlling infection. Antibiotics remain indispensable, particularly in immunocompromised patients at high risk of systemic bacterial infections (Dropulic and Lederman, 2016; Kumar et al., 2022); however, broad-spectrum exposure can disrupt commensal communities, reduce colonization resistance, and promote the expansion of opportunistic pathogens and antimicrobial-resistance genes (Fishbein et al., 2023; Patangia et al., 2022). Consequently, increasing attention has focused on narrow-spectrum and pathogen-selective antibiotics that target specific pathogens while limiting collateral effects on beneficial microbiota (Kumar et al., 2018, 2021a; Rezaei et al., 2025). Nevertheless, conventional broad-spectrum antibiotics can induce persistent ecological alterations and may delay post-antibiotic microbiome recovery (Francino, 2015; Pal Kalia et al., 2023).

Antibiotic stewardship can complement microbiome-preserving approaches by reducing unnecessary antimicrobial exposure. Importantly, recent population-scale evidence linking individual-level prescription data from 14,979 participants with gut microbiome composition demonstrates that antibiotic exposure is associated with measurable alterations in the gut microbial community, providing direct support for the relationship between antibiotic use and microbiome disruption (Baldanzi et al., 2026). Procalcitonin (PCT)-guided algorithms can further reduce unnecessary antibiotic initiation and duration (Dupuy et al., 2013; Lin and Pang, 2018; Schuetz et al., 2011, 2012). However, these clinical outcomes should not themselves be interpreted as evidence of microbiome preservation unless accompanied by direct measurements of microbial diversity and abundance, or post-antibiotic recovery.

In parallel, microbiome-preserving antimicrobial approaches include narrow-spectrum and pathogen-selective agents, which aim to control pathogens while limiting collateral microbiome disruption and resistance selection (Elhag et al., 2022). FimH antagonists targeting adherent-invasive *E. coli* (Poole et al., 2017) and EcoActive, an anti-*E. coli* bacteriophage have also entered clinical investigation (NCT03709628, NCT03808103). These approaches position antimicrobial selection and stewardship within the translational microbiome framework by balancing effective infection control with preservation and recovery of the intestinal microbial ecosystem.

## Bacteriophages as precision antimicrobial platforms

This shift toward ecosystem-preserving antimicrobial stewardship has positioned bacteriophage therapy as a premier platform for executing strain-specific, targeted microbial interventions. Phage-based strategies now encompass naturally occurring lytic phages, engineered phages, CRISPR-delivery phages, fixed phage cocktails, personalized preparations, and microbiome-editing approaches, each with distinct advantages and translational challenges. Their relatively narrow host range compared with conventional broad-spectrum antibiotics may reduce collateral effects on commensal microbiota (Zhao et al., 2026). Naturally isolated lytic phages are being evaluated for multidrug-resistant bacterial infections and targeted modulation of disease-associated gut bacteria (Gómez-Ochoa et al., 2022). Lytic phages directed against *Klebsiella pneumoniae* have shown beneficial effects in preclinical colitis models, while early-phase clinical studies have primarily provided safety and tolerability data (Federici et al., 2022). In parallel, genetically engineered CRISPR-Cas9–armed phages, such as SNIPR001, are being developed to selectively eliminate antibiotic-resistant *E. coli* through targeted genomic disruption (Gencay et al., 2024). Several phage-based approaches are currently under clinical investigation (Table 3).

**Table 3:** Phage-based therapeutics for bacterial infections.

Intervention/sponsor	Pathogen(s) and therapeutic use	Status/phase	Key outcomes/notes	References
TP-122A—TechnoPhage	Mixed pathogens—Ventilator-associated pneumonia	Phase 1–2a	Safety and tolerability assessment	NCT06370598
DUOFAG^®^–MB Pharma	*S. aureus, P. aeruginosa*—Surgical wound infections	Phase 1–2a	Randomized, double-blind, placebo-controlled	NCT06319235
DANCE™ platform—adaptive phage therapeutics	Mixed bacterial pathogens—Diabetic foot osteomyelitis	Phase 1/2	Repeat-dose, multi-site trial; precision phage matching	NCT05177107
AP-PA02—Armata pharmaceuticals	*P. aeruginosa*—RTIs (CF and NCFB)	Phase 2	Inhaled sterile formulation; favorable safety; $5.25M DoD funding	NCT04596319
PhagoDAIR I—Phaxiam therapeutics	*E. coli, P. aeruginosa*—Severe bacterial infections	Phase 1/2	74% infection control after single injection; excellent safety	NCT02116010
SNIPR001/CRISPR-engineered phage—SNIPR Biome	*E. coli*—Infections in malignant patients	Phase 1	First orally dosed CRISPR phage therapy in humans; CARB-X supported	NCT05277350
BX004 Phage cocktail—BiomX	*P. aeruginosa*—Cystic fibrosis	Phase 1	Acquired Adaptive Phage Therapeutics; $50M private placement led by Deerfield and AMR Action Fund	NCT05010577
LBP-EC01/CRISPR-Cas3 phage—Locus biosciences	MDR *E. coli*—UTIs	Phase 2/3	ELIMINATE trial; positive PK and safety; Phase 1b endpoints met	NCT05488340
Personalized phage therapy	*P. aeruginosa*—Chronic otitis media	Case study	Significant bacterial reduction and clinical improvement; local delivery	NCT04191148
TP-102	MRSA—Diabetic foot ulcers	Clinical trial	~70% infection clearance, especially in MDR cases	NCT05948592
Personalized phage therapy	*P. aeruginosa*—Chronic respiratory infection	Case study	Reduced bacterial load; improved lung function; biofilm targeting	NCT05453578
Personalized phage therapy	MDR *A. baumannii*—Sepsis	Clinical trial	Full recovery after failure of all antibiotics	Kumar et al., 2021a
Phage cocktail	*E. coli, K. pneumoniae, P. aeruginosa*—Infected burn wounds	Clinical trial	Reduced infection severity and accelerated wound healing	NCT02116010
Natural phage therapy	MDR *E. coli*—UTIs	Clinical trial	~80% infection clearance; intravesical delivery	NCT03140085
Personalized phage therapy	MDR *K. pneumoniae*—Diabetic foot infection	Case study	Complete resolution after antibiotic failure	Saadaoui et al., 2024
Natural phage therapy	MSSA and MRSA—Chronic rhinosinusitis	Clinical trial	Symptom improvement and reduced colonization in ~60% of cases	ACTRN12616000002482

CF, cystic fibrosis; NCFB, non-cystic fibrosis bronchiectasis; RTIs, respiratory tract infections; MDR, multi-drug resistant; AMR, antimicrobial resistant; PK, pharmacokinetics; MRSA, methicillin-resistant Staphylococcus aureus; MSSA, methicillin-sensitive Staphylococcus aureus; UTIs, urinary tract infections; CARB-X, combating antibiotic-resistant bacteria biopharmaceutical accelerator; I/II, II/III: Phase 1/2, Phase 2/3 clinical trial stages; VAP, ventilator-associated pneumonia.

Although early-phase studies have reported encouraging safety findings, important translational barriers remain, including phage pharmacokinetics and pharmacodynamics, bacterial-density dependence, tissue penetration, neutralizing antibodies, adaptive dosing, host-range determination, phage resistance, gene-transfer risks, manufacturing consistency, and regulatory requirements (Danis-Wlodarczyk et al., 2021; Liu et al., 2021). Advanced delivery approaches, including pH-responsive encapsulation and targeted intestinal-release systems, are being investigated to improve phage stability and local delivery (Hsu et al., 2020). Collectively, these technological advancements position phage therapy as a highly promising platform for personalized microbial interventions; but adequately powered randomized multi-center trials are required to establish clinical efficacy and long-term safety across specific indications.

## Challenges in clinical translation and future horizon

Despite substantial progress, translation of microbiome discoveries into clinical practice remains constrained by interconnected analytical, biological, clinical, manufacturing, and regulatory challenges. A major technical limitation is substantial inter-individual and temporal heterogeneity of the human microbiome [Integrative HMP (iHMP) Research Network Consortium, 2019; Zeng et al., 2026]. Microbiome composition and function are influenced by interacting factors including antibiotic and other medication exposure, diet, age, sex, ancestry, host genetics, geography, lifestyle, socioeconomic conditions, urbanization, disease severity, healthcare access, and baseline microbiota composition, which can contribute to inconsistent microbial signatures and therapeutic responses (Kundu et al., 2017; Zeng et al., 2026; Zmora et al., 2018). Standardized sampling, longitudinal designs (Taheriyoun et al., 2026), careful adjustment for confounding, and mechanistically informed patient stratification are therefore essential for improving reproducibility.

Importantly, microbiome interventions differ substantially in their translational requirements and should not be evaluated using a single framework (Wong, 2026). Donor-derived FMT, standardized microbiota products, conventional probiotics, defined microbial consortia, genetically engineered live biotherapeutics, bacteriophages, and microbiome-derived diagnostics differ in mechanistic certainty, patient selection, dose definition, engraftment, durability, manufacturing, safety, scalability, cost, and regulatory requirements (Table 4). For example, durable engraftment remains challenging for microbial therapeutics (Smillie et al., 2018), whereas engineered living medicines additionally require consideration of genetic stability, circuit failure, environmental shedding, horizontal gene transfer, biocontainment, dose control, manufacturing consistency, and long-term monitoring (Hou et al., 2022; Lynch and Pedersen, 2016). Donor-derived FMT presents different concerns, including donor screening, pathogen transmission, product variability, and immune-related safety (DeFilipp et al., 2019).

**Table 4:** Challenges in translating microbial therapies to clinical applications.

Challenge	Description	Examples/implications	References
Inter-individual variability	Differences in host genetics, diet, lifestyle, and baseline microbiota affect therapy outcomes.	FMT and probiotics show variable efficacy across patients.	Akobeng et al., 2020; Augustine et al., 2023
Microbial engraftment and stability	Difficulty ensuring sustained colonization and function of transplanted or supplemented microbes.	Limited long-term persistence of donor strains after FMT.	Wu et al., 2024
Mechanistic uncertainty	Limited understanding of specific microbial pathways responsible for therapeutic effects.	Hard to predict which strains/metabolites drive clinical outcomes.	Kumar et al., 2024
Safety concerns	Risk of pathogen transfer, unintended immune reactions, or horizontal gene transfer.	FDA reported adverse events in early FMT use.	Chopra et al., 2024
Regulatory complexity	Lack of harmonized guidelines on defining, manufacturing, and approving live biotherapeutics.	Classification differences between FDA (drug/biologic) and EMA (medicinal product).	Philip et al., 2025
Manufacturing and scalability	Challenges in large-scale production, formulation, and storage of live microbes.	Maintaining viability and potency in capsules or lyophilized forms.	Lopetuso et al., 2020
Ethical and patient acceptance	Concerns over FMT “yuck factor,” donor selection, and equitable access.	Patient hesitancy for stool-based therapies.	Wang et al., 2020

Another limitation is the underrepresentation of many global populations in large microbiome datasets. Microbiome composition and function vary with geography and are also shaped by diet, ancestry, lifestyle, socioeconomic conditions, medication exposure, and urbanization (Liang et al., 2026; Lin et al., 2026; Parizadeh and Arrieta, 2023). Consequently, biomarkers and therapeutic strategies developed predominantly in Western industrialized cohorts may show reduced transportability or uncertain effectiveness in underrepresented populations, including those in Africa. Greater global representation and prospective cross-population validation are therefore needed before broadly implementing microbiome-based precision medicine.

Regulatory requirements also differ substantially among therapeutic classes. Donor-derived FMT, standardized microbiota products, live biotherapeutic products, genetically modified organisms, bacteriophages, and microbiome-derived diagnostics require distinct considerations for product characterization, manufacturing, quality control, safety monitoring, and clinical evaluation (Kelly et al., 2015; O'Toole et al., 2017). Regulatory status and guidance should therefore be interpreted using current FDA, EMA, and other authoritative regulatory documents at the stated review cutoff date (Rodriguez et al., 2025). Ethical considerations, including data privacy, ownership of biological samples, equitable access, and representation, also remain important (Metwaly et al., 2025; Ponce de Leon-Derecho and Dable-Tupas, 2025).

Future progress will require more than larger datasets, lower sequencing costs, and increasingly sophisticated computational methods. Priorities include prospective multi-center validation, harmonized clinically meaningful endpoints, standardized manufacturing and quality control, longitudinal assessment of engraftment and durability, long-term safety surveillance, mechanistically informed patient stratification, and adequately powered comparator-controlled randomized trials (Chen et al., 2023; Rodriguez et al., 2025). AI-based approaches may facilitate integration of complex multi-omics data, but their clinical application requires careful attention to data leakage, algorithmic bias, explainability, privacy, external validation, model drift, and prospective clinical evaluation (Chouvarda et al., 2025; Ogut, 2025). Together, these measures will be essential for distinguishing biologically plausible findings from reproducible clinical benefit and enabling responsible translation of microbiome-based diagnostics and therapeutics.

## Conclusion

While the microbiome field remains in the early stages of clinical translation, foundational discoveries are increasingly supporting the development of clinically relevant applications. This progress has culminated in regulatory-approved microbiota-based therapies, with microbiota restoration for selected cases of recurrent *C. difficile* infection representing the most clinically established application. In contrast, microbiome-based interventions for inflammatory, metabolic, neurological, and oncological diseases remain largely investigational, with uncertainties regarding biomarker reproducibility, therapeutic engraftment, safety, manufacturing, and clinical generalizability. Nevertheless, the growing number of clinical trials evaluating microbiome-targeted interventions underscores their potential for precision medicine. Key priorities include standardized methodologies, external biomarker validation, adequately powered randomized trials, durable engraftment assessment, rigorous manufacturing controls, long-term safety monitoring, and globally representative cohorts. As our understanding of complex host–microbiome interactions continues to advance, future research should focus on establishing causal mechanisms, reproducible therapeutic benefits, and long-term safety to support the responsible integration of microbiome-based approaches into routine healthcare.
